# Fucoxanthin induced apoptotic cell death in oral squamous carcinoma (KB) cells

**DOI:** 10.6026/97320630017181

**Published:** 2021-01-31

**Authors:** Petchi Iyappan, M.Devi Bala, M Sureshkumar, Vishnu Priya Veeraraghavan, Arulselvan Palanisamy

**Affiliations:** 1 Senior Lecturer, Faculty of Medicine, Bioscience and Nursing, School of Bioscience, Mahsa University, Saujana Putra Campus, Jalan SP2, Bandar Saujana Putra, 42610, Jenjarom, Selangor, Malaysia; 2 Research Scholar, Muthayammal College of Arts & Science (A Unit of VANETRA Group), Rasipuram, 637408, Namakkal, Tamilnadu, India;; 3 Department of Zoology & Biotechnology, Muthayammal College of Arts & Science (A Unit of VANETRA Group), Rasipuram, 637408, Namakkal, Tamilnadu, India; 4 Department of Biochemistry, Saveetha Dental College, Saveetha Institute of Medical and Technical Sciences, Saveetha University, Chennai - 600 077;; 5 Adjunct Associate Professor,Muthayammal Centre for Advanced Research (MCAR), Muthayammal College of Arts & Science (A Unit of VANETRA Group),Rasipuram, 637408, Namakkal, Tamilnadu, India

**Keywords:** Oral cancer, Antioxidant, Lipid peroxidation, Cytotoxicity, Reactive oxygen species

## Abstract

Fucoxanthin (Fx) is an active compound commonly found in the many types of seaweed with numerous biological activities. The main goal of this investigation is to explore the effect of Fx against the cell proliferation, apoptotic induction and oxidative stress in the oral squamous (KB) cell line. Cytotoxicity of Fx was determined by MTT assay. The intracellular ROS production, mitochondrial membrane potential (MMP) and apoptosis induction in KB cells were examined through DCFH-DA, Rhodamine-123 and DAPI, and dual staining techniques. Effect of Fx on the antioxidant enzymes and lipid peroxidation in the KB cells was studied through the standard procedures. Fx treated KB cells showed morphological changes and reduced cell survival, which is exhibited by the cytotoxic activity of 50 µM/ml (IC50) Fx against the KB cells. The Fx treatment considerably induced the apoptotosis cells (EB/AO) and decreased the MMP (Rh-123) in KB cells. Further, it was pointed out that there was an increased lipid peroxidation (LPO) with decreased antioxidants (CAT, SOD and GSH). These results concluded that Fx has the cytotoxic effect against KB cells and has the potential to induce the apoptosis via increased oxidative stress. Hence, the Fx can be a promising agent for the treatment of oral cancer and it may lead to the development of cancer therapeutics.

## Background:

Oral cancer (OC) is the most predominant carcinoma around the world, which affects many of peoples annually [1]. In head and neck cancer was rare cancer with nasopharyngeal carcinoma noticeable cultural and biological distributions and it have peak of incidences in the world including Southern China and Southeast Asia [2,3]. Additionally, it was advanced disease regionally and concurrent chemoradiotherapy of nasopharyngeal cancer in patients with poor success rate along with many side effects. So, the radiotherapy and surgical techniques have been greatly restricted due to their prognosis remains poor. Even though, prior improvement in analyzing treatment and tumor recurrence rates to reached at 30% of high-risk for OC patients [4]. Now, numerous phytochemicals have been documented as an anti-tumor property to develop the inhibition of cell proliferation and induction of apoptosis that reduces the risk of cancer [5]. Therefore, exploring novel therapeutic approaches for developing therapeutic targets and improving the efficacy of anticancer drugs in nasopharyngeal carcinoma patients is highly beneficial [6]. Fx, a xanthophyll carotenoid is brimming in edible brown seaweeds, it normally polyene chain arranged an allenic bond and oxygenic functional groups. Fx is highly found in the brown algae and the regular consumption of seaweeds may contribute to the anti-cancer effects [7,8]. This property was mostly assisted for several antitumor action of Fx. Fx has a defensive role and displayed anti-progression action in frequent types of carcinoma. Newly, research has assessed the biological activities of epoxy carotenoids as well as Fx in carcinoma cells grown in vitro and illustrated the different cellular point of Fx. The previous researches were highlighted the anticancer potentials of Fx against the colorectal cancer and hepatocarcinogenesis [9,10]. Fx, a nutraceutical [11] showed anti-inflammatory activity [12], antioxidant activity [13,14], neuroprotective activity [15-17], hepatoprotective activity [18,19], nephroprotective activity [20,21], geroprotective activity [22], anti-osteoclastogenic activity [23], antiatherosclerotic activity [24], eryptosis activity [25], antidiabetic retinopathy activity [26], protective effects against thyroid damage [27], antioxidant activity against Subarachnoid Hemorrhage-oxidative stress [28]. The anticancer activity of Fx against different cancers was already reported [29-35]. Fx showed anticancer effects against gastric cancer SGC-7901 cells [36], human cervical cancer cells [37, 38], human glioma cells [39], lung cancer [40-42], human glioblastoma cells [43, 44], breast cancer MCF-7 and MDA-MB-231 cells [45], colon cancer [46-50], human gastric adenocarcinoma MGC- 803 cells [51], human bladder cancer [52-53], colon cancer cells [54,55], liver cancer cells [56-58], human prostate cancer cells [59-62], lymphomas [63, 64], gastric cancer cells [65], human cervical cancer cells [66-68], osteosarcoma [69], human leukemia HL-60 cells [70], anti-sarcoma activity [71], melanoma B16F10 cells [72], leukemia [73,74], and nasopharyngeal carcinoma cells [75]. Therefore, it is of interest to document fucoxanthin induced apoptotic cell death in oral squamous carcinoma (KB) cells.

## Materials and methods:

### Chemicals:

**Figure 1:**
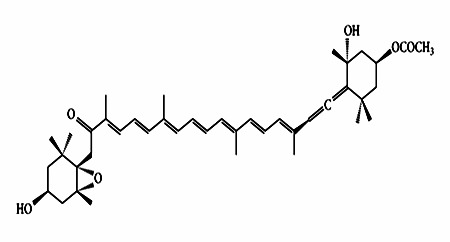
Chemical structure of Fx.

Fucoxanthin (Figure 1) and fetal bovine serum (FBS) was purchased by Sigma Aldrich (USA). Dulbecco's Modified Eagle's Medium (DMEM), phosphate buffered saline (PBS), antibiotics (penicillin and 100 µg/ml of streptomycin), 4,5-Dimethylthiazol-2-yl)-2,5-Diphenyltetrazolium Bromide (MTT) dye, 2',7'-Dichlorofluorescin diacetate (DCFH-DA), trypsin-EDTA, acridine orange (AO/)/ethidium bromide (EB), fetal bovine serum (FBS), rhodamine 123 (Rh-123), ethanol and dimethyl sulfoxide (DMSO) were purchased from HiMedia (USA).

### culture maintenance:

HOC cells (KB) were purchased from the American Type Culture Collection (ATCC), USA, and maintained in a culture medium DMEM supplemented with FBS and antibiotics. Cells were maintained in 5% CO_2_ incubator at 37°C and the experiments were carried out after cell proliferation stage was reached. The nutrient DMEM medium was changed every two days and the production was strictly followed in accordance with the standard procedures.

### Preparation of Fx stock solution:

A stock solution of Fx (1g/L) was prepared in a DMSO (0.5%) and stored at 4°C. From this 2.5, 5, 10, 15 and 20 µl was a pipette out into 1 ml culture media to arrive 25 to 200 µM/ml in each well, respectively.

### Treatment of the KB cells:

The oral cancer (KB) cells were maintained as a monolayer at 37°C in a humidified atmosphere of CO_2_ (5%) in DMEM medium containing heat-inactivated FBS and antibiotics. Following trypsinization, the HOC cells (KB) were serially subculture using a trypsin/EDTA. After 70 to 80% confluence achievement, prior to treatment, the cancer cells were starved for 24 h in growth medium. Fx was suspended in DMSO, to make a stock solution, aliquot and stored at -20°C. Time response studies were conducted to determine the IC50 values.

### Cytotoxicity assay:

The cytotoxic effect of Fx against cell growth of KB cells was assessed by the way of Mosmann et al. (1983) [76]. Cancer cell line (KB) was seeded in the 96 well plates. After, treatment with the different doses Fx (25 to 200 µM/ml) cells was incubated for 24 h at 30°C in a CO_2_ incubator. MTT dye was added to each well at the dose of 10 mg/mL and KB cells were again incubated for 4 h at 37°C. Followed by the incubation, the medium removed and 100 µl of DMSO was added to the each well to dissolve the formazan crystals. The absorbance was measured at 490 nm (Microplate reader, Bio-Rad). The half maximal inhibitory concentration (IC50) values were calculated and the optimum doses were analyzed at various time duration. The inhibitory concentration dose (IC50) is the number of cells able to inhibit cell proliferation by 50%, which was calculated graphically for each well growth curve.

### Measurement of intracellular reactive oxygen species (ROS):

Overnight grown cells were seeded in the 6 well plates and incubated for 24 hour at 37°C along with the different doses of (25 and 50 µM/ml) Fx. After incubation, cells were rinsed with PBS by centrifugation and loaded with 20 µM DCFH-DA in DMEM and incubated for 30 min at 37°C. Afterward, treated cells were rinsed with DMEM and fluorescent level was assessed every 5 min in over 30 min (excitation 485 nm, emission 535 nm) using a spectrofluorimetry at 37°C.

### Measurement of mitochondrial membrane potential (MMP):

The effect of Fx in the MMP disruption level in the KB cells was assessed using the Rh-123 staining, which is a lipophilic cationic fluorescent probe for mitochondria. The cells were incubated along with the Fx at various doses (25 and 50 µM/ml) for 24 h at 37°C. Rh-123 at the final dose of 10 µg/ml was added to each well and then KB cell line was again incubated for 30 min at 37°C in a CO_2_ incubator. Subsequently the KB cells were cleansed with PBS and the fluorescence was examined under a fluorescence microscope using a blue filter.

### Measurement of apoptotic induction using AO/EB staining:

The fluorescence microscopic analysis of apoptotic cell death was determined by dual staining. Cancer cells (KB) were seeded at 5 x 104 cells per well in a tissue culture plate (6 well) and incubated for 24 hour. Followed by the treatment with IC50 dose of Fx for 24 h the KB cell line were detached (trypsin/EDTA), rinsed through PBS and then stained with a mixture of AO/ EB (1:1 ratio) at 37°C for dark room 5 min. The stained cells were examined through a fluorescence microscope at 40x magnifications.

### Estimation of LPO and antioxidant enzymes level:

The KB cells were harvested after the 24 h treatment with Fx (20 and 25 µM/ml) and subsequently subjected to biochemical assessments. The status of LPO in Fx treated KB cells were examined through measuring LPO (lipid peroxide) byproduct of TBARS (Thiobarbituric acid) reactive substance, and the levels of enzymatic antioxidants such as catalase (CAT) [77], superoxide dismutase (SOD) [78], the intracellular enzyme of glutathione peroxidase (GSH) level was measured [79], respectively. The untreated well was employed as a control for all assays, respectively.

### Statistical analysis:

The results are expressed as mean ± SD of triplicate measurements. The statistical comparisons were achieved by one-way ANOVA, followed by the DMRT using SPSS version 17.0 software. The results were considered statistically significant if the p 7lt; 0.05.

## Results:

### Inhibition of oral cancer KB cells growth by Fx:

**Figure 2:**
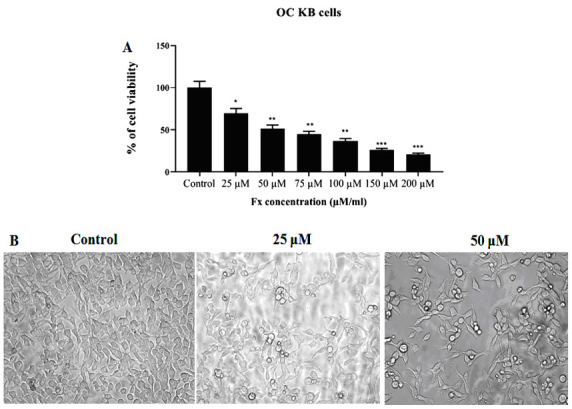
Effect of Fx on cell viability and morphological characteristics of OC cells by MTT assay. (A) Depicts Fx treated with cancer cells at various concentrations (Control, 25 to 200 µM/ml) respectively. B) Morphological changes in control and Fx treated KB cells for 24 hr. Results are expressed as cancer cells treatment with either control or Fx for 24 h. Values were presented as mean ± SD of asterisks independent experiments ANOVA followed by DMRT. Asterisks indicate statistically different from control *p < 0.05.

Figure 2 shows the chemical structure of Fx. The effects of Fx treatment at 25 to 200 µM to the KB cell viability were measured by the MTT assay. Fx treatment notably suppressed the cell proliferation of the KB cells after 24 h of treatment (Figure 2A). 50% of viable cells were observed at the dose of 50 µM on KB cells for 24 h. From this study the IC50 value of Fx were measured as 50 µM. The survival of KB cells were diminished notably in a dose dependent manner with an IC50 (the absorption causing 50% live and dead cells) value at 50 µM/ml. Fx resulted in the irregular morphology of KB cells and also possessed the cell shrinkage, rounded form, and reduced the viability of KB cells (Figure 2B).

### Effect of Fx on the intracellular ROS levels in the KB cells:

**Figure 3:**
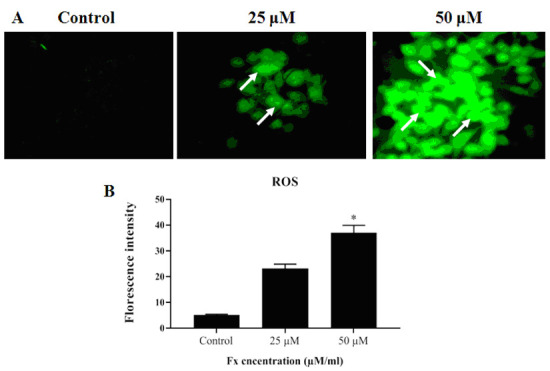
Effect of Fx on the intracellular ROS levels in the KB cell. (A) Fluorescence microscopic showing the production of intracellular ROS using DCFH-DA staining in OC cells (KB). White arrow mark represents clearly visible DCF fluorescence in cancer cells treated with Fx in various concentration manners. (B) Intracellular ROS examined by spectrofluorometer. The values were presented as mean ± SD of asterisks independent experiments ANOVA followed by DMRT. Asterisks indicate statistically different from control *p < 0.05.

A significant enhancement in the intracellular ROS formation was observed in Fx treated KB cells. Fx treatment (25 µM) significantly induced the ROS generation in KB cells. Photomicrographs (Figure 3A) clearly showed the intense green fluorescence due to ROS generation in the control KB cells. Fx treated (25 µM) KB cells were illustrated weak background of green fluorescence. The treatment with Fx (25 and 50 µM/ml) revealed the increased ROS creation as revealed through augmented DCF dye fluorescence in the nucleus of KB cells (Figure 3B).

### Effects of Fx on the level of MMP in the KB cells:

**Figure 4:**
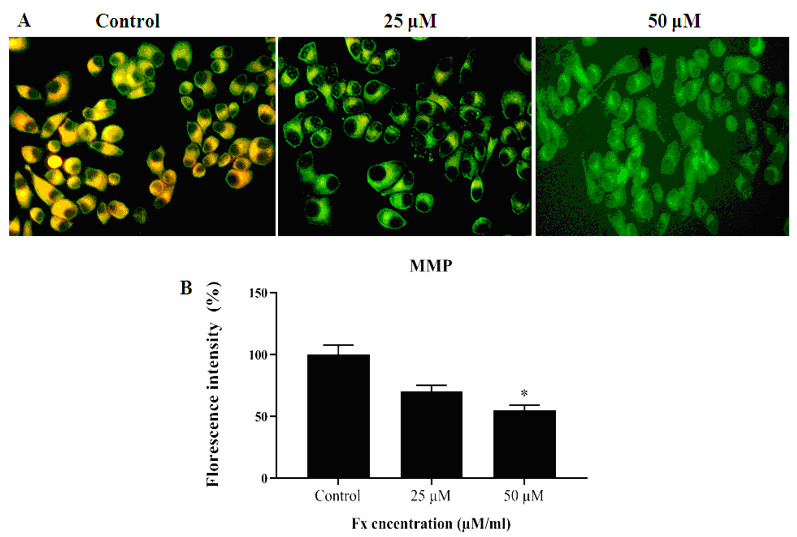
Effects of Fx on the level of MMP in the KB cells. (A) Effect of Fx on the MMP of HOC cells (KB). OC cells were treated with various concentrations Fx for 24 h, stained with Rh-123 and the mitochondrial depolarization patterns of cancer cells were observed. Results the gradual decrease of red/green fluorescence indicates a decrease MMP in a various concentration manner were investigated by fluorescent microscope. In the fluorescent image shows control (Rh accumulation); Fx (25 and 50 µM/ml) (No Rh-123 accumulation). B) Quantification of MMP in the spectrofluorometry. Values are given as mean ± SD of three experiments in each concentration ANOVA followed by DMRT. Asterisks indicate statically different from control * p < 0.05.

MMP was analyzed by using the Rh-123 staining after 24 h exposure of KB cells to the different doses of (25 and 50 µM/ml) Fx. The fluorescent (Rh-123) dye ratio was found, as confirmation by decreased intensity of the red and green fluorescence ratio (Figure 4A). The turn down in MMP was concentration dependent of Fx, when compared to the negative control. Fluorescence images (Figure 4B) represent the buildup of Rh-123 dye from orange red to green fluorescence as compared to the control and the gathering found to be diminished in Fx treated cancer cells (KB).

### Effects of Fx on the induction of apoptosis in the KB cells:

**Figure 5:**
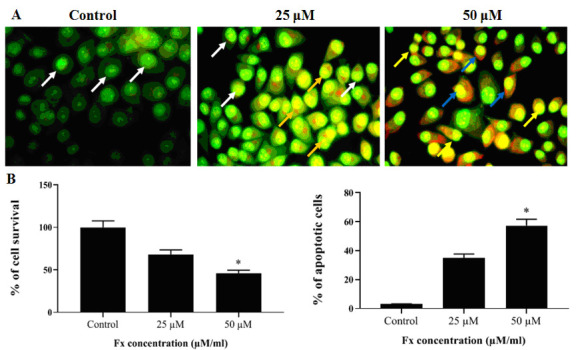
Effects of Fx on the induction of apoptosis in the KB cells. (A) OC cells (KB) treatment within control and Fx at different doses at 24 h, stained with AO/EB and then evaluated by fluorescence microscopy. White arrow indicates green florescence; Orange arrow indicates apoptotic bodies; Blue arrow indicates apoptotic cells; Yellow arrow indicates necrotic cells. Fx induced apoptosis by generating ROS and interruption of MMP. (B) % of apoptotic cells were measured by scoring apoptotic and viable cells (KB). The values are given as mean ± SD of three experiments in each group ANOVA followed by DMRT. Asterisks indicate statistically different from control * p < 0.05.

The effect of Fx treatment on the induction of apoptosis in the KB cells was confirmed through the morphological fluctuations after AO/EB staining were examined (Figure 5A). AO stained to the cells show the green fluorescence after intercalation into DNA in viable cells. EB stained cells shown red fluorescence when injured cell membrane integrity in the KB cells. Apoptotic morphological appearance of some of the chromatin condensation, alterations in the size, nuclear fragmentation and the shape of KB cells, as examined through fluorescence microscopic, were measured predominantly after Fx treatment for 24 h. KB cell nuclei treated with Fx concentration 25 & 50 µM/ml maximum increase in the quantity of apoptotic cells as observed respectively related to the control (Figure 5B).

### Effect of Fx on LPO and antioxidants levels in KB cells:

**Figure 6:**
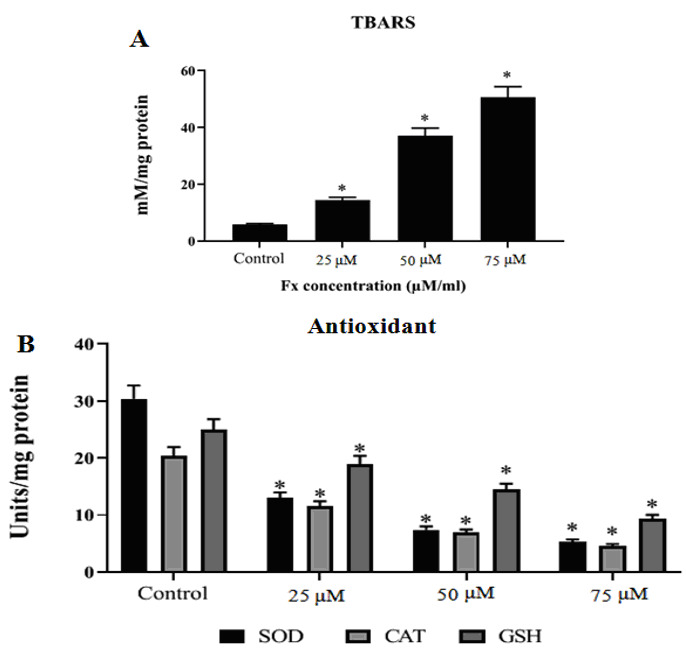
Effect of Fx on LPO and antioxidants levels in KB cells. Fx induced LPO and modulates cellular antioxidant levels in HOC cells (KB). The values are given as mean ± SD of three experiments in each group ANOVA followed by DMRT. Asterisks indicate statistically different from control * p < 0.05.

Levels of TBARS decreased significantly in control cells (Figure 6A). Interestingly, Fx treated (50 µM) KB cells depicted progressively elevated status of TBARS as compared with control KB cells. Figure 6B shows the levels of antioxidants i.e. SOD, CAT and GSH in the normal and Fx treated KB cells. The levels of antioxidant enzymes level were significantly increased in the control KB cells. Treatment with Fx (25 and 50 µM/ml), the levels of antioxidants were notably decreased in KB cells as compared to control cells (Figure 6B).

## Discussion:

To the best of our knowledge, this study is the first to reveal the in vitro cytotoxic and apoptosis inducing activities of Fx in the HOC cells (KB). The Fx treatment dose dependently decreased the cell viability of oral cancer KB cells, which demonstrating that the Fx might be the active antitumor agent as observed in this study. The numerous natural compounds were studied scientifically for their anticancer activity against various cancers, which may lead to the development of promising anticancer agents [80]. MTT assay commonly used technique that was carried out to assess the cytotoxic activity of sample agents, ability of tetrazolium salt (MTT) into an insoluble formazan product were decreased due to the mitochondrial dehydrogenase found in live cells [76]. MTT assay were assisted to confirm the cytotoxicity as well as supports the dose related cell toxicity effect of Fx on the KB cells (Figure 2). The cytotoxic effect of Fx were observed in the KB cells, which indicates the Fx possessed increased cytotoxicity to the oral cancer KB cells. Our findings were coincides with the previous work [56]. Liu et al. (2009) [58] has proved the strong cytotoxic effect of Fx at different dose and their anti-proliferative effect against the SK-Hep-1 cells for 24 h. Conversely, the suppressive effect was similar for concentrations >1 µM after 48 h. Fx represses the tumor formation via an enhancing gap functional intercellular communication, a variety of machinery, arresting the cell cycle at G1/G0 and inducing cell death. The intracellular ROS synthesis in the cells leads to the oxidative stress and leads to apoptosis. When increases ROS formation, morphological changes undergoes and gives late apoptotic modulators were appeared by AO/EB staining in edited study [81]. In the present finding, the increased amount of ROS formation by Fx treatment at various concentrations (25 and 50 µM/ml) was noted as compared to untreated KB cells. Recent studies were reported that the pro-oxidant actions of Fx with other carotenoids assisted for the induction of apoptosis in HOC. Nevertheless, the similar apoptotic inducing activity of Fx in promyelocytic leukemia cell lines were found but from their results in H_2_O_2_ resistant cell lines finally have suggested that ROS is not the mainstream pathway for cell death affected [74]. Opposing to this study, Kim et al. (2010) [70] have experientially proved the inhibition in leukemia cell lines growth by Fx and further they have credited to help ROS generation by Fx that leads to apoptosis. The alteration of the level of MMP and its modification is the target point to identify the cancer condition. When compare the cancer cells with normal cell, displays the moderately diminished inactive MMP level [82]. MMP abnormalities were examined by the accumulation of Rh-123 fluorescence dye in KB cells treated with various concentrations of Fx. Administered with Fx found in the developed depolarization of the MMP as retrieved by the emitted fluorescence intensity for Rh-123 absorption compared to the untreated cells. We observed the uptake of Rh-123 in the mitochondrial region of normal cells. In Fx treated KB cells not appeared Rh-123 accumulation. This result was indicates that the MMP was changed during by the Fx treatment. So that, we demonstrated that the Fx stimulated decrease of MMP may due to the ROS synthesis, which can encourage the MMP and following initiation of apoptosis [18]. Cell death is a useful pattern that was distinguished by apoptotic morphological structures and development of DNA damage [83]. Consequently, towards regulates whether the enhances suppressive abilities of Fx resulted in the earlier development of cell damage and the morphological arrangement of HOC cells were identified using AO/EB staining assay to produce cell apoptosis. Figure 5A clearly showed the early stage apoptotic cells with yellow color and late stage apoptotic cells with orange color in KB cells nuclei treated with Fx at 25 µM/ml. The maximum increase in the quantity of apoptotic cells was observed at 50µM/ml concentration of Fx treated cells respectively related to control. The intracellular ROS secretion in the cells can be augmented by the excessive free radicals, and it can be scavenged by the cellular antioxidant enzymes such as SOD and CAT [84]. Fx suppressed the intracellular ROS level in the KB cells. Current study revealed that the orally administered Fx demonstrates the modulated amount of LPO enhanced the antioxidant in DEN-induced liver cancer [10]. Fx has been prevented and also protected the neurotoxicity induced by Aβ1-42 in cerebral cortex neuron in SH-SY5Y cells [17]. Fx may be contestant for possible use in cancer diagnosis and therapy were validated in U251 human glioma cells death by stimulating ROS-induced oxidative damage and dysfunction of MAPKs and PI3K/AKT pathway [43]. Ye et al. (2017) [38] reported that Fx in in vitro and in vivo xenograft experiments demonstrated that the combination of TRAIL with Fx showed synergistically inhibitory effects on cervical cancer cells. Primary treatment of Fx were diminished LDH elevation and cytosolic ROS content, further increased intracellular reduced GSH and further they studied Fx 50 µM were saved against the oxidative damage in a non-dose dependent manner, with the optimal effects, finally they recommended that Fx have been protects the cells affects by H_2_O_2_ induced oxidative damage in L02 cells via the PI3K-dependent activation of Nrf2 signaling mechanism [85]. Recently reported that the compound isolated from Undaria pinnatifida (Wakame) were suppresses cell growth and movement in human LEC further inhibited the malignant phenotype in human breast cancer cells and lymph angiogenesis [86]. These consequences were recommended that Fx in restrain tumor stimulate by lymph angiogenesis cellular and experimental model, which highlighting its potential use as an anti-lymphangiogenic agent for anti-tumor metastatic comprehensive therapy in patients with breast cancer. Pangestuti et al. (2013) [87] have been evaluated that Fx induces anti-inflammatory and anti-oxidant effects in amyloid-β42 -induced BV2 microglia cells, as indicated by the decreased expressions of pro-inflammatory cytokines and ROS formation. Temporarily, Fx notably suppressed LPO in PC-12 cells under oxidative stress situation, while the powerful anti-inflammatory and anti-oxidant properties of Fx was associated with the diminished iNOS/NO pathway, go together with the inhibition of TNF-a and IL-6 protein pattern 4) [88]. Currently, the usage alternative herbal-based medicines are increased extensively. Conversely, a lot of people are in doubt to use such drugs they are not active drug scientifically or their mechanism of action is not properly known [89]. Hence, look for finding out secure, reasonable and well-organized natural plant products that are experimentally confirmed to be successful and are non-toxic, because most of the anti-cancer drugs used in cancer therapy are toxic and have adverse side effects. Many studies has proved the efficacy of phyto compounds amongst numerous originate in crude plant extract is significant for diagnosis and therapeutic purposes [90, 91]. Even though the effect of Fx was currently been analyzed over an in vitro HOC, it is very similar that the current data were founded can be exhibited in animal or in human. Though, to examine more analysis might be approved out on in vivo animal models, which will hopefully be taken up in the next phase of our program using mice model.

## Conclusion

We document data on the fucoxanthin induced apoptotic cell death in oral squamous carcinoma (KB) cells for further consideration.
